# Necrotizing amebic colitis in an elder patient: an unexpected autopsy finding

**DOI:** 10.4322/acr.2023.456

**Published:** 2023-11-27

**Authors:** Maria Aparecida Marchesan Rodrigues, Bruno Miamoto, Rosa Marlene Viero

**Affiliations:** 1 Universidade Estadual Paulista (UNESP), Departamento de Patologia, Faculdade de Medicina de Botucatu, Botucatu, SP

**Keywords:** Intestinal *Entamoeba histolytica* infection, Diagnosis, Death Cause

## Abstract

Necrotizing amebic colitis is an uncommon amebiasis complication associated with high mortality. We present a case of necrotizing amebic colitis in an old patient whose diagnosis was revealed at postmortem examination. An 81-year-old man died at home without medical attention. The postmortem examination revealed ulcers involving the entire colon and intestinal perforation. The ulcers were large, geographic, and necrotizing, extending from the cecum to the rectum. The histological examination disclosed the infectious etiology by showing amebic trophozoites at the base of the ulcers. No extra-intestinal lesions were found. No information about previous episodes of dysentery or travel could be obtained. The potential role of aging or drug-causing immunosuppression and the evolution of chronic and latent intestinal infection to a severe and invasive form of amebiasis is discussed. This case reinforces the value of postmortem examination for diagnosing diseases not clinically identified.

## INTRODUCTION

Amebiasis, a human infection of the large intestine by the protozoan parasite *Entamoeba histolytica*, affects approximately 10% of the general population, ranking as the second leading cause of death related to parasitic diseases.^1-3^ It is an important cause of diarrhea globally, especially in developing countries with inadequate hygiene and sanitation conditions.^1-4^ Most infections are asymptomatic, but 10% to 20% progress to amebic colitis and liver abscesses.^1,2,4,5^ Involvement of the lung and brain has been reported in disseminated amebiasis.^6^ If recognized and treated timely; amebiasis is a curable disease.^7-10^ However, missed diagnosis may lead to potentially severe complications like intestinal perforation, peritonitis, hemorrhage, strictures, or obstruction.^4,7,11^ Amebic liver abscesses occur in 3-9% of cases of intestinal amebiasis.^1,2,5^ Acute fulminant necrotizing amebic colitis is an uncommon complication. It is associated with high mortality, even if recognized and treated appropriately.^10,12,13^ Immunosuppression, pregnancy, and corticotherapy are risk factors associated with fulminant disease.^1,4,13-15^

Although endemic in Brazil, there is a lack of national reports about the burden of amebiasis. Many cases are probably under-reported, especially those from the northern regions. Similarly, information on the mortality related to *E hystolitica* infection is scarce in Brazil. Therefore, we present a case of fulminant amebic colitis in an old patient from a Brazilian southeast State, in which the diagnosis was established at post-mortem examination. The potential role of aging or medication in the course of a chronic to severe and invasive form of amebiasis is discussed.

## CASE REPORT

An 81-year-old man was found dead at home and was submitted to post-mortem examination at the local coroner. The patient was from Southern Brazil in São Paulo state. He was a retired lawyer, married, but living alone. No information about previous episodes of dysentery, medical therapy or travel history could be obtained.

**Figure 1:**
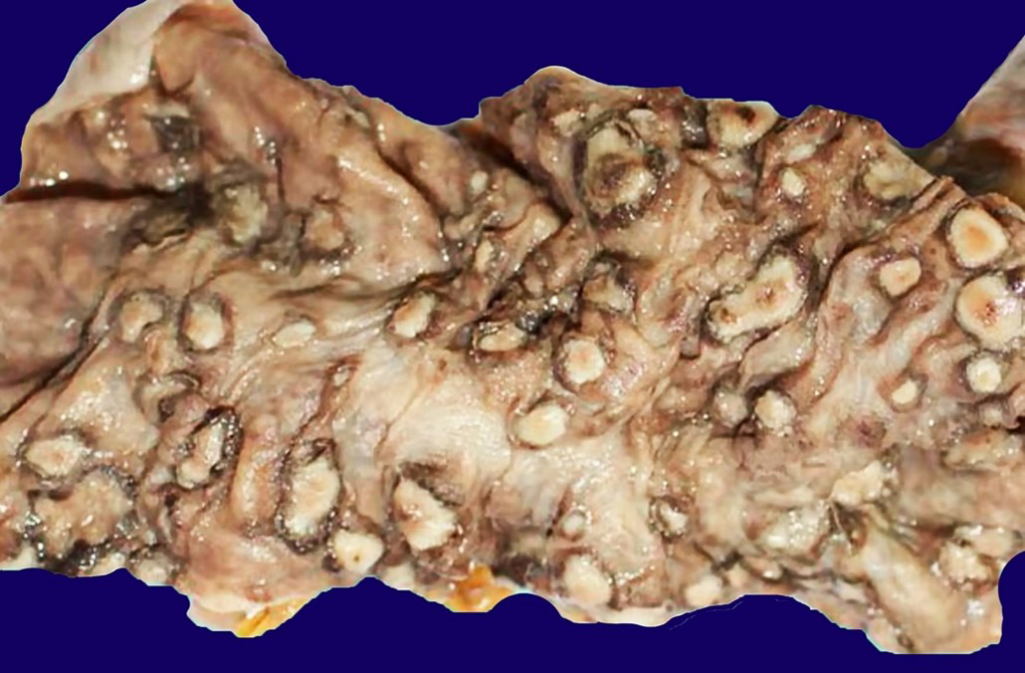
Gross findings of the colon revealing multiple necrotizing lesions with well-defined edges.

The post-mortem examination disclosed a malnourished patient weighing 50 Kg and measuring 165 cm. At the opening of the abdominal cavity, there was 1,400 mL of serosanguineous ascites. The gross examination of the gastrointestinal tract revealed the involvement of the entire colon by many necrotizing lesions and ulcers, sometimes with a flask-shaped appearance extending from the cecum to the rectum (Figure 1).

**Figure 2:**
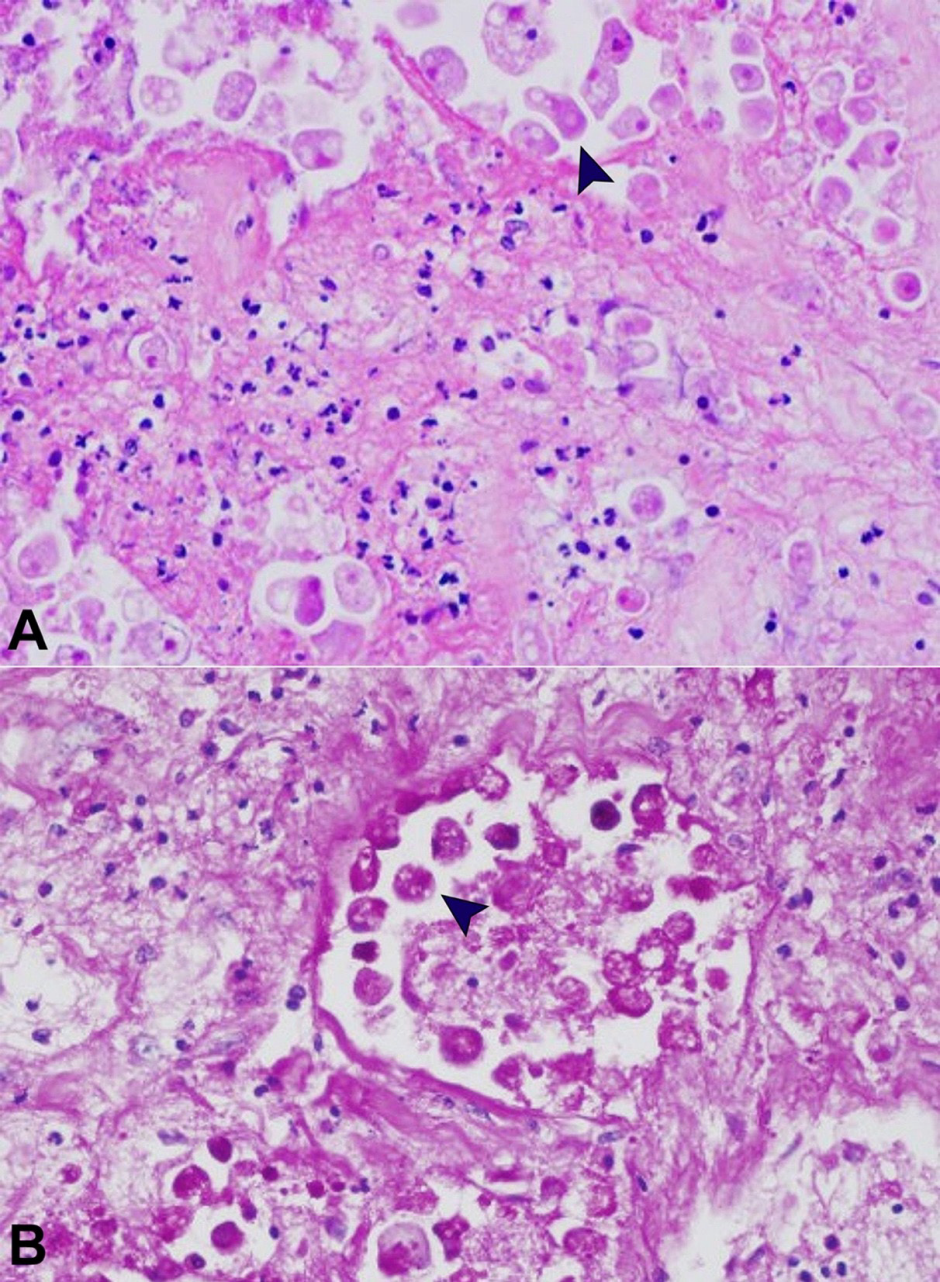
Photomicrographs of the colonic ulcers. **A** **–** showing amebic trophozoites with foamy cytoplasm with round eccentric nuclei (arrowhead) resembling macrophages. (H&E, 400X); **B**  **–** Periodic Acid-Schiff (400X) (arrowhead).

An intestinal perforation was observed in the right colon together with local acute suppurative peritonitis. Histologically, the ulcers were deep, extending into the submucosa and undermining borders. The lesions comprised necrotic material with prominent nuclear debris and few inflammatory cells. Amebic trophozoites were found at the ulcers’ edges, together with mild mononuclear inflammation (Figure 2A). Amebic trophozoites had a foamy cytoplasm with round eccentric nuclei resembling large macrophages. Red blood cells were phagocytized by the trophozoites, which were stained by Periodic Acid-Schiff (PAS) (Figure 2B). There was no hepatic, pulmonary, or other extra-intestinal involvement. Death was attributed to sepsis from the intestinal lesions.

## DISCUSSION

Amebiasis remains an important enteropathogen worldwide. It is a leading cause of diarrhea globally, especially in tropical and subtropical regions where inadequate hygiene and poor sanitary conditions prevail.^2-4^ In Brazil, amebiasis accounts for high morbidity in many regions.^16-21^ A recent meta-analysis showed a significant prevalence and variable distribution percentages of *E histolytica* infection among Brazilian regions, with the highest prevalence in the north (28.9%) contrasting to 1.1% in the south and 0.3% in the southeast regions.^21^ In this report, the patient was from a city in the southern region of São Paulo state, a Brazilian developed area.^22^ This region boasts a Human Development Index of 0.8, along with good standards of sanitation and hygiene resources.^22,23^

The hallmark of amebic colitis is a parasitic invasion into the intestinal mucosa and submucosa. *E histolytica* trophozoites produce cysteine proteinases that are responsible for the invasion and inflammation of the colonic mucosa.^1^ The lesions occur predominantly in the caecum and ascending colon; however, any part of the large bowel may be involved. The gross findings of amebic ulcerated lesions may be similar to those observed in inflammatory bowel disease.^2,8,24^

In the current case, many ulcers were found in the colon. They were geographic and necrotizing, measuring between five and two centimeters and extending from the cecum to the rectum. The histological examination showed an extension of the necrosis and inflammation to the muscular layers in many areas. The involvement of the whole large intestine, as observed in the present case, is sporadic and associated with high mortality.^10,25^ The microscopic examination was crucial to disclose the infectious etiology by showing round organisms with 20-30 mm and intracytoplasmic erythrocytes at the ulcers’ edge, recognized as amebic trophozoites, confirming the diagnosis of invasive amebiasis. Although the three species of the entamoeba complex: *Entamoeba histolytica*, *Entamoeba dispar*, and *Entamoeba moshkovskii,* cannot be distinguished by morphology, the context of the lesions in the present case are highly suggestive of *Entamoeba hystolytica* as the etiologic agent. However, it should be pointed out that under certain circumstances, the “avirulent” species of *Entamoeba díspar* and *Entamoeba moshkovskii* can become pathogenic and cause disease.^26-28^

The guidelines for autopsy pathology have recognized the need for microscopic examination in medical autopsies. A study conducted at two French University Hospitals revealed that in 30% of cases where macroscopic autopsy findings failed to identify a specific lesion as the cause of death, microscopic examination provided insights into the etiology of the death.^29^ Similar values of discrepancies between the macroscopic and histologic diagnosis at necropsy for lesions in the lung (38.7%), liver (35.1%), and kidney (30.3%) were reported in a Brazilian study.^30^ In this setting, the Royal College of Pathology^31^ recommends a systematic microscopic examination of the vital organs, and The College of American Pathologists^32^ favors microscopic examination of organs with macroscopic lesions. Therefore, our report reinforces the importance of microscopic examination in medical autopsies to determine of the cause of death.

It is also essential to consider that Entamoeba’s morphologic features may mimic tissue macrophages or ganglion cells, and the diagnosis may be missed if the pathologist is unaware of this resemblance. Ancillary tests like Periodic-acid Schiff (PAS) stain or immunohistochemistry can be used to depict Entamoeba cysts and/or trophozoites in the lesions.^11,33^

The clinical manifestations of amebic colitis are variable. The most common gastrointestinal symptoms include watery or bloody diarrhea and abdominal pain.^1,2,7,9,34^ However, intestinal symptoms may be misinterpreted as other inflammatory bowel diseases, especially in areas of low prevalence of amebiasis.^8,11,34^ In this case, the information about the clinical symptoms was unavailable.

Various risk factors are associated with the development of fulminant amebic colitis, including male gender, age over 60 years, concomitant liver abscess, malnutrition, immunosuppressive diseases, or immunosuppressive therapy.^8,13^ In this report, the patient was an octogenarian man in agreement with the higher prevalence of invasive amebiasis among men than in women and with the immune system decline related to aging.^35-37^ Both innate and adaptive T and B-immunity present reduced activity with aging.^36,37^ Since protection against amebiasis mainly relies on cell-mediated responses,^38^ it is conceivable, in this case, that immunosenescence may have contributed to the transition from a chronic latent intestinal infection to an invasive form of amebiasis.

Another possibility that can be taken into account is the use of some medications, such as corticosteroids. Shirley and Moonah^13^ reported fulminant amebic colitis in twenty-four patients treated with high doses of systemic corticosteroids. We do not have information on any drug our patient could have taken. Apart from the patient's age and gender, no additional risk factors such as prior travel or medication usage could be identified. Therefore, in this case, impairment of host immune defenses due to aging or some medication combined with the tissue damage caused by the parasite may have promoted the invasive outcome of amebic infection.

In conclusion, this case of extensive amebic colitis in an old patient from a Brazilian-developed region highlights the importance of histological examination in the workup of medical autopsies for the diagnosis of diseases not clinically identified.
